# Research on the impact of college students’ “Buddha-like” characteristics on social responsibility and the mediating role of social support

**DOI:** 10.3389/fpsyg.2025.1691896

**Published:** 2025-12-05

**Authors:** Zhengnan Qi, Fang Han

**Affiliations:** 1 Zhejiang Agricultural Business College, Shaoxing, Zhejiang, China; 2 National Science Library, Chinese Academy of Sciences, Beijing, China

**Keywords:** college students, “Buddha-like” characteristics, social responsibility, social support, dimensional heterogeneity

## Abstract

**Introduction:**

Against the backdrop of China’s social transformation, the “Buddha-like” mindset has permeated the college student population, emerging as a subcultural phenomenon that serves as both a stress buffer and a potential developmental risk.

**Methods:**

Grounded in social cognitive theory, this study administered a questionnaire survey to 1,147 undergraduate and vocational college students. Utilizing structural equation modeling (SEM), hierarchical regression, and Bootstrap mediation tests, it systematically investigated the pathways through which “Buddha-like” Characteristics influence social responsibility and examined the mediating role of social support.

**Results:**

The findings revealed that: (1) “Buddha-like” Characteristics can be aggregated into a four-dimensional structure comprising Low Goal Commitment, Low Self-Transcendence, Low Interpersonal Communication, and High Dependence, which overall demonstrated a significant negative association with Social Responsibility; (2) Social Support played a partial mediating role between them, with the indirect path through Family Support being the most prominent; (3) The influence of “Buddha-like” Characteristics exhibited dimensional heterogeneity: Low Goal Commitment had both direct and indirect effect paths; Low Self-Transcendence exhibited a significant suppression effect, where Social Support masked its underlying negative nature; Low Interpersonal Communication exerted its influence entirely indirectly through Social Support; (4) High Dependence displayed characteristics of “Inconsistent Mediation,” reflecting its dual functionality in family versus broader social contexts. Furthermore, the study found that “Buddha-like” Characteristics were more pronounced among undergraduates, students majoring in humanities, sciences, and agriculture, as well as those without Student Positions or Honor Statuses.

**Discussion:**

This research constructs an integrated model, provides new insights into the complex effects of the “Buddha-like” mindset, and identifies the practical pathway of mitigating its negative effects by strengthening social support, particularly Family Support.

## Introduction

“Buddha-like” characteristics, as one of the most popular “internet slang” terms, have rapidly spread and gained widespread acceptance in online spaces, media communication, and daily social interactions. As the term “Buddha-like” gradually extends from the internet into reality, the specific psychological tendencies it represents have transcended simple online expressions and have started to be widely used as a combined concept to label different groups. In some specific social groups, “Buddha-like” tendencies have even to some extent integrated into their life philosophies, becoming a behavioral guideline in their daily practices (Cheng and Qi, 2024; Xu and Liu, 2022), thus forming a distinctive “Buddha-like” cultural phenomenon (Yan et al., 2024; Li, 2019). Currently, the “Buddha-like” phenomenon is rapidly spreading among some youth groups. Some college students identify themselves as “Buddha-like youth,” using attitudes such as “having no desires and no demands” and “letting things take their course” as benchmarks, which manifest in a lack of goals and a pursuit of comfort, among other characteristics (Cheng and Qi, 2024; Xu and Liu, 2022). These behaviors stand in stark contrast to the struggle spirit required by Social Responsibility. Therefore, how to change the “Buddha-like” tendencies of college students and stimulate their fighting spirit is a direction worth exploring in depth.

Since the term “Buddha-like” became popular among youth, academia has paid close attention and conducted research on various aspects, such as the characteristics (Yan et al., 2024), formation mechanisms (Li, 2019; Bu et al., 2018; Wu and Zhang, 2021), influences (Song, 2018; Sun and Liu, 2022), and countermeasures (Sun et al., 2018) of “Buddha-like youth.” Most existing research tends to discuss the relevant issues of “Buddha-like youth” from a macro structural perspective, while studies from a micro or meso perspective, focusing on the youth subject, are relatively scarce. In-depth analysis using quantitative indicators of various “Buddha-like” Characteristics in college students, exploring the effects of these characteristics on Social Responsibility, is even more limited.

Based on the above analysis, this study, grounded in social cognitive theory, uses 1,147 valid samples from both undergraduate and vocational colleges as research objects. The study measures their performance in four “Buddha-like” characteristic dimensions: Low Goal Commitment, Low Self-Transcendence, Low Interpersonal Communication, and High Dependence (Xu and Liu, 2022). It also introduces Social Support as a mediating variable, measuring the mediating effect of Social Support between college students’ “Buddha-like” Characteristics and Social Responsibility through three dimensions: Family Support, Friend Support, and Other Support. The research is of significant importance for revealing college students’ “Buddha-like” ideology, formulating effective policies to curb the adverse effects of “Buddha-like” culture, and guiding college students to establish a positive mindset.

## Theoretical foundation and research hypotheses

### Social cognitive theory

Social cognitive theory, as one of the major theories in fields such as education and social psychology, analyzes the interaction of three factors—individuals, environment, and behavior—to study and explain human social behavior. This theory posits that individual factors, environmental factors, and behavioral factors mutually influence and determine each other. Specifically, there is an interaction mechanism between these three elements: (1) the individual plays a dominant role in their behavior, and the outcomes of their behavior affect the individual’s subjective emotions, leading to self-regulation; (2) the environment not only shapes an individual’s personality traits but also influences their subjective emotional changes, while the individual’s subjective cognition can also impact the surrounding environment; (3) behavior is both a means for individuals to change and adapt to the environment and is also determined by the environment’s influence on the intensity of behavior in reality (Bandura, 1986). In addition to the triadic reciprocal determinism, social cognitive theory also encompasses key concepts such as self-efficacy, outcome expectations, and observational learning. Specifically, driven by subjective agency, individuals form corresponding expectations and action plans by evaluating themselves and the environment, actively influencing the environment through self-management to achieve the desired goals. Throughout this process, individuals continuously reflect on themselves based on the external environment, adjusting their self-efficacy evaluations to form new expectations, self-management, and behavioral performance (Bandura, 2001). Research has found that an individual’s perception of their ability to control their behavior is influenced by both personal characteristics (such as past experiences, physical and mental states, observing others, and persuasion from others) and external environmental factors. With increased self-efficacy, individuals are more likely to face challenges when difficulties arise (Bandura, 2012).

### Research on “Buddha-like youth”

The term “Buddha-like” originated from a Japanese magazine in 2014, which referred to young men who showed little interest in changing their existing lifestyle or situation and were more focused on their own hobbies as “Buddha-like” men (Miao, 2019). With the spread of the term on the internet, China first used “Buddha-like” in 2014 to describe a specific group of people, and by the end of 2017, driven by a Weibo article, the term became a popular social topic, subsequently expanding into terms like “Buddha-like youth,” “Buddha-like girls,” and “Buddha-like parents,” reflecting the existential reality and psychological state of certain groups (He, 2024). In light of the widespread discussion on the “Buddha-like” phenomenon, research has initiated preliminary studies on this phenomenon within youth groups, including college students, and has produced corresponding results. Regarding its essence, some scholars believe that the “Buddha-like” phenomenon among the youth is closely related to the development of the times and social reforms, serving as a form of collective expression with a mild form of resistance. It is seen as a subcultural manifestation stemming from the “Losing Culture” prevalent in contemporary society (Bu et al., 2018; Zhang, 2018). Other scholars assert that the “Buddha-like” attitude of college students is essentially a manifestation of modern-day cynicism, with individuals adopting indulgence, lack of faith, value confusion, self-mockery, and other behaviors as a form of escapism or decadence (Zhu, 2018; Ye, 2018).

Regarding the features of the “Buddha-like youth,” some scholars, through descriptive phenomena and theoretical deduction, have identified characteristics such as non-competition, worry-free, seeing things in a detached manner, and indifference to outcomes (Dai and Yang, 2018). Other scholars, through grounded research and quantitative analysis, have found that “Buddha-like youth” exhibit four characteristics: low goal commitment, low self-transcendence, low interpersonal communication, and high dependence (Xu and Liu, 2022). Building on this, further studies have explored the impact of the “Buddha-like” attitude on individual responsibility and the consequences it may bring. Research has shown that the “Buddha-like” attitude can lead to the loss of core beliefs, the erosion of moral values, the dissolution of traditional social roles, and the disintegration of a sense of mission (Song, 2018; Sun and Liu, 2022). Specifically, the “Buddha-like” attitude among youth reflects a weakening of social responsibility (Tong, 2023), and this phenomenon greatly impacts the sense of social responsibility, national consciousness, and national mission of college students and other youth groups. It may cause the collapse of mainstream societal values and lead to a broader loss of social responsibility (He, 2024). Therefore, the “Buddha-like youth” culture not only affects college students’ daily behavior and decision-making but also reconstructs or undermines their personal values and social responsibility.

Furthermore, as a subculture, the “Buddha-like” culture establishes its own discourse system, logic, and behavioral norms, thereby making the boundaries between “Buddha-like” college students and other social “circles” more distinct. This trend leads to stronger social isolation and a shrinking and segregating pattern of social interactions (Liu, 2023). Existing studies suggest that youth influenced by “Buddha-like” and other “Losing Culture” often exhibit more closed-off social relationships, lacking adequate social support (Xu and Liu, 2022; Fu, 2024). Consequently, reduced social interactions lower the level of social support for “Buddha-like” college students. Despite the relatively weak level of social support among “Buddha-like” college students, social cognitive theory suggests that individuals, in addition to their subjective agency, also continuously reflect on themselves based on external environments and adjust their self-efficacy evaluation to form new expectations, self-management, and behavioral performance (Bandura, 2001). Therefore, Social Support, as an environmental factor in the social context (Wu et al., 2006), can not only motivate individuals but also strengthen their commitment to goals and enhance their execution (Furrer and Skinner, 2003). For example, a study by Schepers et al. (2008) investigating college students demonstrated that support from teachers and peers fosters psychological safety and strengthens the sense of team responsibility. Similarly, Sun et al. (2018) found that social support and its three dimensions have a significant positive correlation with medical students’ sense of social responsibility.

This study hypothesizes that under the influence of the “Buddha-like” culture, when college students believe, their actions will not lead to change or they feel they lack the ability to solve problems, they may experience low self-efficacy. As a result, they may adopt a “Buddha-like” attitude (such as avoiding proactive change, maintaining the status quo) to reduce pressure and avoid failure, and choose behaviors that appear to require less effort and risk. This leads to a reluctance to actively engage in social activities or take on social responsibilities. However, as social individuals, college students will still form and maintain some positive, lasting social relationships to enhance their sense of security and belonging (Martin and Dowson, 2009), which can then promote self-regulation, helping them expand their range of behavioral choices and become more actively involved in social activities and assume social responsibilities.

### Theoretical boundaries of the “Buddha-like” construct

Having clarified the origins, manifestations, and basic characteristics of “Buddha-like youth,” it is necessary to situate the “Buddha-like” construct within a broader conceptual map and systematically differentiate it from related constructs such as Passive Coping, Cynicism, and Amotivation to deepen the understanding of its theoretical connotations. Although these concepts share superficial similarities in behavioral presentations like “low proactivity,” the “Buddha-like” construct is rooted in the specific subcultural context of China’s social transition period (Bu and Jia, 2020). Its theoretical origins, internal structure, and psychological mechanisms demonstrate unique theoretical boundaries. Clarifying these boundaries is a theoretical prerequisite for advancing research on the “Buddha-like” phenomenon and ensuring the validity of its measurement.

1. Distinction from Passive Coping

Passive Coping is rooted in stress-cognition interaction theory, and its core refers to short-term, context-specific adaptive strategies adopted by individuals to manage immediate emotions or problems when facing specific stressful events (Lazarus and Folkman, 1984). In essence, “Buddha-like” characteristics are fundamentally different, as they do not constitute an immediate reaction to specific stressors. Instead, they represent a cross-situationally stable life attitude and subcultural identity. Their formation stems from a strategy of self-withdrawal adopted by individuals in the context of tense societal public-private relationships and a high-pressure, high-risk social environment (Zhao, 2019).

In terms of structural characteristics, Passive Coping typically manifests as a single emotion-focused coping strategy (e.g., distraction, emotional suppression). It focuses on temporarily alleviating the negative emotions induced by stress rather than changing the stressor itself, presenting a “situation-triggered—singular response” behavioral pattern, often confined to a regulatory approach of “single strategy—immediate emotional buffering” (Bonanno and Burton, 2013). Conversely, “Buddha-like” Characteristics manifest as a multidimensional integration of attitudes—specifically, “Low Goal Commitment, Low Self-Transcendence, Low Interpersonal Communication, and High Dependence” (Xu and Liu, 2022). Their influence permeates various aspects, including life attitudes, social interaction patterns, and self-expectations.

Therefore, Passive Coping is essentially a “short-term, situational response to specific stress,” whereas the “Buddha-like” construct constitutes a “stable, personalized mindset system permeating multiple life domains.”

1. Distinction from Cynicism

Cynicism is rooted in Social Critical Theory. Its core lies in individuals, after perceiving the gap between ideology and reality, adopting a stance of “superficial compliance but internal denial” to cope with reality. It is essentially a skeptical compromise with mainstream values (Li, 2022). In fundamental contrast, the “Buddha-like” mindset is more moderate and detached, characterized by “de-emphasizing the importance of values rather than negating the values themselves.” As Ou (2021) pointed out, the entertaining and self-mocking mechanisms of “Buddha-like” make it a stress-buffering strategy, rather than a value confrontation characteristic of Cynicism.

Regarding the formation mechanism, Cynicism stems from a dual drive of “external trust breakdown – internal cognitive rigidity”: externally, it is often triggered by factors like Psychological Contract Violation or lack of Organizational Justice; internally, “Learned Apathy” forms through attributional bias (Su et al., 2025). Overall, it belongs to a passively triggered psychological defense. In contrast, the formation of “Buddha-like” characteristics is more an active process of psychological adjustment by the individual. It is a self-initiated adaptation achieved through “goal downgrading” and “emotional relief” in the face of structural pressures (Bu and Jia, 2020), essentially an active construction of meaning regulation.

In terms of impact effects, Cynicism has a clear negative orientation, significantly negatively predicting individual well-being and organizational performance, and eroding the foundation of social trust (Su et al., 2025). The impact of “Buddha-like” Characteristics, however, presents a dialectical nature: while it somewhat inhibits innovative behavior, it contributes to maintaining emotional stability and interpersonal harmony, functioning as a “psychological safety valve” in high-pressure environments (Yan et al., 2024).

Therefore, Cynicism is essentially a “negative compromise system dominated by value skepticism,” whereas the “Buddha-like” construct is an “active mindset framework oriented towards stress adaptation.”

1. Distinction from Amotivation

Amotivation is rooted in self-determination theory. Its core refers to a non-autonomous psychological state wherein individuals completely lack the intention and willingness to act due to an inability to perceive the connection between behavior and outcomes (Ryan and Deci, 2000). Fundamentally distinct, “Buddha-like” characteristics do not represent a fundamental lack within the motivational system. Rather, they constitute a life philosophy of “low obsession and going with the flow” actively chosen by individuals in high-pressure social contexts, representing a cross-situationally stable subcultural mindset and identity (Yan et al., 2024).

Regarding the formation mechanism, Amotivation arises from the dual obstruction of external environmental pressure and internal psychological needs: externally, from factors like imbalanced resource distribution and controlling management (Wang et al., 2024); internally, from the frustration of basic psychological needs such as Autonomy, Competence, and Relatedness (Guo et al., 2024). This ultimately leads individuals into a passively triggered state of motivational exhaustion. In contrast, the emergence of “Buddha-like” Characteristics is an active process of psychological adjustment undertaken by the individual. It is a voluntary choice made in the face of social structural dilemmas, achieving psychological compensation and anxiety relief through “downplaying obsessions” (Bu and Jia, 2020).

In terms of structural characteristics, amotivation demonstrates a “singularly negative” behavioral pattern: cognitively dismissing the value of activities with Low Self-Efficacy, and behaviorally exhibiting comprehensive withdrawal characteristics such as perfunctory work engagement and avoidance (Wang et al., 2024; Guo et al., 2024). Conversely, “Buddha-like” Characteristics manifest as an integration of multidimensional attitudes, specifically “Low Goal Commitment, Low Self-Transcendence, Low Interpersonal Communication, and High Dependence” (Xu and Liu, 2022), or alternatively contextualized as a four-dimensional structure of “indifference and contentment with the status quo” in workplace settings (Yan et al., 2024). Notably, these behaviors maintain clear boundaries (Xu and Liu, 2022)—while not pursuing extraordinary achievements, individuals nevertheless fulfill their fundamental tasks and responsibilities.

Regarding impact effects, Amotivation is consistently associated with negative outcomes, significantly positively predicting job burnout and negatively predicting job satisfaction (Guo et al., 2024). In comparison, the influence of “Buddha-like” Characteristics demonstrates dialectical nature: while negatively affecting employee creativity, it simultaneously promotes work-related well-being (Yan et al., 2024), and may even function as a “safety valve” for conflict mitigation (Cheng and Qi, 2024).

Therefore, Amotivation essentially constitutes a “passive state of motivational deficiency resulting from environmental and psychological blockages,” whereas the “Buddha-like” construct represents an “actively constructed, cross-domain stable mindset system.”

In summary, the “Buddha-like” Characteristics defined in this study constitute a composite, culturally contextualized, and multidimensional construct that integrates low proactivity, low sociability, and high situational compliance. It is distinct from stress-driven Passive Coping, value-denying Cynicism, and intention-deficient Amotivation. This theoretical delineation not only clarifies the independence and novelty of the “Buddha-like” construct but also provides a clear conceptual foundation and measurement guidance for subsequent empirical research.

### “Buddha-like” characteristics and social responsibility

Currently, the definition of “Buddha-like” characteristics is largely based on individual passive stress coping styles and stress response patterns. Previous studies have shown that “Buddha-like” characteristics are mostly manifested as passive characteristics such as avoidance, resignation, submission, and retreat (Cao and Zeng, 2020). Subsequently, Xu and Liu (2022), through the development of a “Buddha-like” characteristics scale for college students and applying exploratory and confirmatory factor analysis, found that college students’ “Buddha-like” characteristics can be divided into Low Goal Commitment, Low Self-Transcendence, Low Interpersonal Communication, and High Dependence, presenting a “three lows, one high” characteristic. Specifically, Low Goal Commitment reflects the lack of commitment to goals and the corresponding goal-pursuing behavior, Low Self-Transcendence reflects the lack of the will to meet challenges or break through the current self, Low Interpersonal Communication indicates social withdrawal and poor interpersonal skills, and High Dependence indicates the lack of autonomy, conforming behavior, and lack of principles. Many studies have confirmed that individuals with prominent “Buddha-like” characteristics often display avoidance of challenges, shirking of responsibilities, and self-deprecating humor, using these to escape the pressure of social responsibility (Fu, 2024).

Social responsibility is generally defined as the attitude and behavioral tendency of an individual to actively take actions for the common good of society and its members from an altruistic standpoint. It requires not only the recognition and understanding of these duties and obligations but also an active concern and service for the social good through actions (Carroll, 1991). Unlike general responsibility, social responsibility has clear personal characteristics, focusing on how an individual recognizes and identifies the duties and obligations assigned by society through their own beliefs and specific actions (Meng, 2019). Related studies have found that social and cultural contexts and self-efficacy have a significant impact on the social responsibility of college students (Shen and Li, 2020). For example, Sun (2021) proposed that youth subcultures, as a form of “counterculture,” are centered on passive values that conflict with socially dominant norms and values, which can weaken college students’ sense of social responsibility. A study by Sun et al. (2018) and others found that there is a significant positive correlation between self-efficacy and social responsibility. Based on this, this study hypothesizes that “Buddha-like” characteristics negatively associated with social responsibility and proposes the following hypotheses:

*H1*: “Buddha-like” Characteristics are negatively associated with Social Responsibility;

*H1a*: Low Goal Commitment is negatively associated with Social Responsibility;

*H1b*: Low Self-Transcendence is negatively associated with Social Responsibility;

*H1c*: Low Interpersonal Communication is negatively associated with Social Responsibility;

*H1d*: High Dependence is negatively associated with Social Responsibility.

### “Buddha-like” characteristics and social support

Social support is a multidimensional concept, including both objective material and emotional support as well as the individual’s subjective perception and use of such support (Cohen and Wills, 1985). It is generally defined as an individual’s access to tangible material support and emotional support from their social network (family, friends, teachers, etc.) (Yuan et al., 2023). Since social support tends to function through social interactions, it can be equated to the provision and exchange of interpersonal relationships, and is influenced by individuals’ subjective perceptions of different types of support (Thoits, 2011). Moreover, there are differences in personality characteristics, perceptions, and behaviors of both the providers and recipients of social support, which affect the quantity and quality of social support received (Williamson and O'Hara, 2017). In a study of emergency department nurses in a tertiary hospital, Wu et al. found a significant positive correlation between self-efficacy and social support, meaning that the higher the self-efficacy, the stronger the perception of social support (Wu et al., 2020). Similarly, Fang’s research showed that self-efficacy in vocational college students had a significant positive correlation with social support (Fang, 2022).

According to Xu and Liu’s (2022) research, “Buddha-like” college students maintain a certain distance from interpersonal relationships and social interactions (Low Interpersonal Communication), and are more likely to show dependence and submission in these interactions (High Dependence). They typically display lower frequency and depth of social interactions, which to some extent reduces their participation in social activities. Additionally, “Buddha-like” students tend to have a smaller and more homogeneous social circle, preferring to associate with people who share similar values and lifestyles. However, social support arises from interactions between individuals and others, meaning that “Buddha-like” students tend to perceive less support within their social network. Therefore, Low Goal Commitment and Low Self-Transcendence in “Buddha-like” Characteristics are expressions of Low Self-Efficacy. Based on this, this study hypothesizes that “Buddha-like” Characteristics negatively associated with Social Support and proposes the following hypotheses:

*H2*: “Buddha-like” Characteristics are negatively associated with Social Support;

*H2a*: Low Goal Commitment is negatively associated with Family Support;

*H2b*: Low Goal Commitment is negatively associated with Friend Support;

*H2c*: Low Goal Commitment is negatively associated with Other Support;

*H2d*: Low Self-Transcendence is negatively associated with Family Support;

*H2e*: Low Self-Transcendence is negatively associated with Friend Support;

*H2f*: Low Self-Transcendence is negatively associated with Other support;

*H2g*: Low Interpersonal communication is negatively associated with Family Support;

*H2h*: Low Interpersonal communication is negatively associated with Friend Support;

*H2i*: Low Interpersonal communication is negatively associated with Other Support;

*H2j*: High Dependence is negatively associated with Family Support;

*H2k*: High Dependence is negatively associated with Friend Support;

*H2l*: High Dependence is negatively associated with Other Support.

### Social support and social responsibility

Although research on social support and social responsibility is relatively scarce, some scholars have found that individuals with higher levels of social support exhibit more prosocial behavior (Gest et al., 2001), which is an important factor in ensuring the development of individual responsibility (Jia et al., 2020). Ahn’s (2016) study found that social support directly affects the social responsibility of Korean adolescents, with social support enhancing their sense of social responsibility. Similarly, Yeun’s et al. (2023) study concluded that there is a significant positive correlation between social support and social responsibility. Furthermore, social support may influence social responsibility through various paths. Compared to objective social support, subjective social support can more effectively reflect an individual’s internal psychological experience and is more predictive of behaviors such as prosocial actions (Haber et al., 2007; Chou, 2000). Thus, Zhao and Zhang’s (2006) research indicated that there is a close relationship between individual responsibility development and the perception of social support. Cheng et al. (2003) also found that the more social support an individual perceives, the stronger their willingness to participate in social activities, which enhances their social responsibility. Based on this, this study hypothesizes that each dimension of Social Support positively associated with social responsibility and proposes the following hypotheses:

*H3*: Social Support is positively associated with Social Responsibility;

*H3a*: Family Support is positively associated with Social Responsibility;

*H3b*: Friend Support is positively associated with Social Responsibility;

*H3c*: Other Support is positively associated with Social Responsibility.

### The associative mediating role of social support

Social Cognitive Theory posits that personal, environmental, and behavioral factors mutually influence one another (Bandura, 1986). Therefore, in the association between “Buddha-like” characteristics and social responsibility, social support acts as an important environmental factor that may be linked to enhancing self-efficacy and positive social behavior, thereby being associated with higher social responsibility. Based on the theoretical analysis above, it can be inferred that social support plays a buffering role in the association between “Buddha-like” characteristics and social responsibility, i.e., social support may be associated with a reduction in the negative links between “Buddha-like” characteristics and social responsibility. For individuals with “Buddha-like” characteristics, social support provides a protective mechanism, making them feel more confident and capable when facing social responsibilities. This protective mechanism, through emotional comfort, information guidance, and material assistance from family, friends, teachers, and other social networks, helps individuals better cope with the challenges of social responsibility and promotes their positive social behavior. Based on this, this study hypothesizes that each dimension of Social Support plays a mediating role in the association between each dimension of “Buddha-like” Characteristics and Social Responsibility, and proposes the following hypotheses:

*H4*: Social Support plays a mediating role in the association between “Buddha-like” Characteristics and Cocial Responsibility;

*H4a*: Family Support plays a mediating role in the association between Low Goal Commitment and Social Responsibility;

*H4b*: Friend Support plays a mediating role in the association between Low Goal Commitment and Social Responsibility;

*H4c*: Other Support plays a mediating role in the association between Low Goal Commitment and social responsibility;

*H4d*: Family Support plays a mediating role in the association between Low Self-Transcendence and social Responsibility;

*H4e*: Friend Support plays a mediating role in the association between Low Self-transcendence and social responsibility;

*H4f*: Other Support plays a mediating role in the association between Low Self-Transcendence and social responsibility;

*H4g*: Family Support plays a mediating role in the association between Low Interpersonal Communication and Social Responsibility;

*H4h*: Friend Support plays a mediating role in the association between Low Interpersonal Communication and Social Responsibility;

*H4i*: Other Support plays a mediating role in the association between Low Interpersonal Communication and Social Responsibility;

*H4j*: Family Support plays a mediating role in the association between High Dependence and Social Responsibility;

*H4k*: Friend Support plays a mediating role in the association between High Dependence and Social Responsibility;

*H4l*: Other support plays a mediating role in the association between High Dependence and Social Responsibility.

## Research design

### Data collection

This study employed a cross-sectional survey design. Data collection was conducted intensively over a one-week period in January 2023 using a convenience sampling method. The sample selection aimed to deeply explore the “Buddha-like” mindset among university students in regions with developed socio-economics and concentrated higher education resources. Consequently, it primarily covered 12 undergraduate and vocational colleges in eastern and coastal provinces of China, specifically including Zhejiang, Guangdong, and Shandong provinces. This sampling strategy ensured that the research subjects effectively reflected the core phenomenon under study, with the sample exhibiting good internal heterogeneity in terms of institution type, major field, and urban development level.

The sampling and recruitment procedures were as follows: the research team coordinated with course instructors, counselors, and homeroom teachers from the participating institutions. Electronic questionnaire links were uniformly distributed within the classes under their supervision. The questionnaire was generated via a professional online survey platform and disseminated through channels such as class WeChat groups and class QQ groups.

#### Questionnaire retrieval and quality control

A total of 2,586 questionnaires were distributed. Through rigorous screening of the returned data (excluding questionnaires with obvious patterned responses, contradictory answers to reverse-coded items, or excessively short completion times), 1,147 valid questionnaires were ultimately obtained, yielding an effective response rate of 44.3%. To quantitatively assess the potential impact of non-response bias on sample representativeness, this study compared the final valid sample included in the analysis with the total returned sample on several core demographic characteristics. Chi-square test results indicated no statistically significant differences in the distribution between the valid sample and the total returned sample regarding Gender (*χ*^2^ = 1.752, *p* = 0.186), Student Position (*χ*^2^ = 0.038, *p* = 0.846), and Honor Status (*χ*^2^ = 3.539, *p* = 0.060). This result suggests that the data screening process did not introduce systematic bias, and the final valid sample adequately represents the initial respondent group in terms of key demographic structure, thereby confirming that non-response bias poses limited interference to the core conclusions of this study.

#### *A priori* power analysis

To ensure the ability to detect the anticipated small effect size in the study’s core model (i.e., the comprehensive mediation model including control variables, dimensions of “Buddha-like” characteristics, dimensions of social support, and social responsibility), an *a priori* power analysis was performed using G*Power 3.1 software. The analysis employed the “Linear multiple regression: Fixed model, *R*^2^ increase” test, suitable for assessing the significance of overall effects composed of multiple predictor variables (e.g., mediation effects). Parameters were set following strict standards: effect size *f*^2^ = 0.02, significance level *α* = 0.05, and statistical power (1–*β*) = 0.95. The calculation results indicated that the minimum sample size required to test the study’s model was 1,146. The final valid sample size for this study was *n* = 1,147, which aligns almost perfectly with this requirement, sufficiently demonstrating that the current sample size provides solid statistical power support for testing the mediation effects in this study.

#### Ethical compliance statement

Throughout the design and implementation process, this study strictly adhered to the ethical principles of the Declaration of Helsinki (2013) and the Guidelines for Ethical Research in Chinese Social Sciences (2021). Prior to submission, to obtain formal ethical approval, the authors submitted a detailed description of the ethical procedures to the institutional research ethics administration. The office issued an Ethical Compliance Certificate on May 16, 2025, explicitly confirming the study’s full compliance with ethical norms. In practice, all ethical standards were implemented during the data collection phase: all participants were required to actively confirm their consent before completing the questionnaire, were clearly informed of the research purpose, the anonymity and confidentiality of the data, and knew they could withdraw unconditionally at any time. No personally identifiable information was collected throughout the questionnaire process. All data were stored on encrypted servers compliant with China’s National Information Security Level 3 Protection standards. This study did not involve any physiological or psychological interventions or high-risk procedures. The distribution of the surveyed sample is presented in Table 1.

**Table 1:** Sample distribution (*n* = 1,147).

Indicator	Category	Frequency	Percentage (%)
Gender	Male	467	40.71
	Female	680	59.29
Grade	Freshman (1st year)	463	40.37
	Sophomore (2nd year)	306	26.68
	Junior (3rd year)	162	14.12
	Senior (4th year)	138	12.03
	Graduate	78	6.80
School category	Undergraduate college	611	53.27
	Vocational college	536	46.73
Major	Humanities	228	19.88
	Science	217	18.92
	Engineering	133	11.60
	Agriculture	39	3.40
	Arts	79	6.89
	Business and Management	400	34.87
	Other	51	4.44
Student position	Yes	542	47.25
	No	605	52.75
Scholarship status	Yes	415	36.18
	No	732	63.82
Honor status	Yes	541	47.17
	No	606	52.83
Place of origin	Rural	522	45.51
	Urban	388	33.83
	Town	237	20.66

Due to rounding, the sum of percentages for each variable may not equal 100%.

### Variable measurement

To ensure that the data better reflects the actual situation of the survey subjects, this study adopts mature measurement scales from both domestic and international sources, with individual modifications to fit the specific context of this study.

#### Measurement of “Buddha-like” characteristics

The measurement scale used is based on the “Buddha-like” Characteristics scale for college students developed by Xu and Liu (2022), which includes four dimensions: Low Goal Commitment, Low Self-Transcendence, Low Interpersonal Communication, and High Dependence, with a total of 20 items. The operational definitions and example items of each dimension are detailed in Appendix A. The scale uses a Likert 5-point method to calculate the overall mean score of the questionnaire. A higher score indicates a more pronounced “Buddha-like” Characteristic in college students. In this study, the Cronbach’s *α* coefficients for Low Goal Commitment, Low Self-Transcendence, Low Interpersonal Communication, and High Dependence are 0.882, 0.831, 0.849, and 0.703, respectively. The overall Cronbach’s α coefficient for the “Buddha-like” Characteristics scale is 0.919.

#### Measurement of social support

The measurement scale used is the Perceived Social Support Scale developed by Blumenthal et al. (1987), which includes three dimensions: Family Support, Friend Support, and Other Support, with a total of 12 items. The scale uses a Likert 5-point method to calculate the overall mean score of the questionnaire. A higher score indicates more Social Support received by the college students. In this study, the Cronbach’s *α* coefficients for Family Support, Friend Support, and Other Support are 0.885, 0.881, and 0.858, respectively. The overall Cronbach’s *α* coefficient for Social Support is 0.938.

#### Measurement of social responsibility

The measurement of Social Responsibility is based on the conscientious dimension of the Big Five Personality scale, which is a unidimensional scale with 12 items (Zhang et al., 2021). The scale uses a Likert 5-point method to calculate the overall mean score of the questionnaire. A higher score indicates a higher level of social responsibility among college students. In this study, the Cronbach’s α coefficient for Social Responsibility is 0.921.

### Research method

This study utilized SPSS 27.0, Amos 26.0, Mplus 8.3, and the SPSSAU online statistical analysis platform for data processing and model testing. The analytical procedure followed these steps:

1. Common Method Bias Test

To mitigate potential biases arising from common method variance, procedural controls were implemented during the questionnaire design stage (e.g., ensuring anonymity, item rearrangement). During the statistical testing phase, Harman’s single-factor test was employed for initial screening. Furthermore, Confirmatory Factor Analysis was conducted using the unmeasured Latent Method Factor Approach recommended by Podsakoff et al. (2003) to rigorously assess the severity of common method bias.

1. Measurement Model Test

First, Confirmatory Factor Analysis (CFA) was performed using Amos 26.0 to evaluate the reliability and validity of the measurement scales. Convergent validity was assessed by calculating composite reliability (CR) and the average variance extracted (AVE). Discriminant validity was examined by comparing the goodness-of-fit of different factor structure models (e.g., an eight-factor model vs. other competing models), supplemented by the Heterotrait–Monotrait (HTMT) ratio of Correlations Criterion.

1. Measurement Invariance Test

To ensure the validity of subsequent comparisons based on grouping variables (e.g., School Category, Student Position), this study employed Mplus 8.3 to conduct Multi-Group Confirmatory Factor Analysis (MGCFA). This involved sequentially testing for configural invariance, metric invariance, and scalar invariance.

1. Descriptive Statistics and Correlation Analysis

SPSS 27.0 was used to calculate the means and standard deviations of all key variables and to perform Pearson correlation analysis, providing a preliminary exploration of the relationships between variables. Simultaneously, independent samples t-tests and one-way analysis of variance (ANOVA) were used to examine differences in core variables across demographic variables.

1. Hypothesis Testing

The main effects and mediating effects were tested using two complementary statistical methods:

**Structural equation modeling (SEM) analysis.** Amos 26.0 was used to construct a two-second-order structural equation model incorporating the second-order factors of “Buddha-like” Characteristics and Social Support, in order to examine the overall action pathways among the variables. The Bootstrap Method (with 5,000 resamples) was employed to calculate the confidence intervals for the overall mediating effect of Social Support.

**Hierarchical regression and multiple mediating effect analysis.** To precisely test the specific mediating pathways through which each dimension of “Buddha-like” characteristics influences Social Responsibility via each dimension of Social Support, this study used the SPSSAU platform. Controlling for relevant variables, the analysis specified 4 independent variables (the four dimensions of “Buddha-like” characteristics), 3 mediating variables (the three dimensions of Social Support), and 1 dependent variable (Social Responsibility), and conducted a parallel multiple mediation effect analysis (using Bootstrap sampling with 5,000 resamples).

## Data analysis and hypothesis testing

### Common method bias assessment

Given that “Buddha-like Characteristics,” “Social Support,” and “Social Responsibility” are all subjective perception variables of the respondents, this study adopted a combined strategy of “procedural controls + *post hoc* statistical tests” to minimize the potential interference of Common Method Bias (CMB) to the greatest extent. The specific procedures are as follows:

1. Procedural Controls

During the questionnaire design and data collection stages, the following measures were implemented to reduce bias at the source:

Questionnaire items used clear and concise wording, avoiding ambiguous statements and professional jargon to ensure respondents accurately understood the questions.

The questionnaire structure and logic were optimized, with items arranged in an order progressing “from easy to difficult” and “from factual to attitudinal,” to reduce careless responding due to fatigue.

Respondents were explicitly informed that the questionnaire was “anonymous” and that “data would be used solely for academic research,” with assurances of information confidentiality, to mitigate social desirability bias.

1. *Post Hoc* Statistical Tests

To systematically assess the severity of common method bias, this study employed three complementary statistical methods, applied progressively according to their level of stringency:

Stage 1: Harman’s Single-Factor Test (Preliminary Screening).

An unrotated exploratory factor analysis (principal component analysis) was conducted on all 44 measurement items, focusing particularly on the variance explained by the first extracted factor. The results showed that the variance explained by the first unrotated principal component was 31.473%, which is below the commonly used threshold of 40%, providing preliminary indication that severe common method bias is not present in this study.

Stage 2: Single-Factor Confirmatory Factor Analysis (Further Verification).

Considering the relatively low sensitivity of Harman’s single-factor test, this study further employed confirmatory factor analysis (CFA) to examine the fit of a Single-Factor Model (where all items were forced to load onto a single latent factor). The results indicated an extremely poor fit for this Single-Factor Model (CFI = 0.52, TLI = 0.50, RMSEA = 0.12; see the “Single-Factor Model” row in Table 2), falling far short of the widely accepted fit standards in the field. This provides strong evidence against the presence of pervasive common method bias.

Stage 3: Unmeasured Latent Method Factor Approach (Core Verification, based on ULMC logic).

**Table 2:** Assessment of common method bias.

Factor model	CFI	GFI	TLI	IFI	RMSEA	RMR
1. Single-factor model	0.52	0.40	0.50	0.52	0.12	0.14
2. Measurement model	0.89	0.84	0.88	0.89	0.06	0.05
3. Common factor model	0.89	0.89	0.92	0.92	0.05	0.03
Difference (model 3–model 2)	0.00	0.05	0.04	0.03	0.01	0.02

For a more rigorous test, this study adopted the Unmeasured Latent Method Factor Approach recommended by Podsakoff et al. (2003). Specifically, based on the theoretical Multi-Factor Measurement Model (the baseline model), an unmeasured common method factor uncorrelated with all theoretical latent constructs was introduced. All measurement items were allowed to load simultaneously onto their corresponding theoretical latent construct and this common method factor, thereby constructing a rival model.

A comparison of the fit indices between the two models (see Table 2) revealed that the improvements in fit indices for the model incorporating the common method factor, compared to the baseline measurement model, were as follows: ΔCFI = 0.00, ΔGFI = 0.05, ΔTLI = 0.04, ΔIFI = 0.03, all below the critical threshold of 0.10; the reductions in RMSEA and RMR were 0.01 and 0.02, respectively, both below 0.05.

According to established academic standards, these minimal differences indicate that incorporating the common method factor did not lead to a significant improvement in model fit, confirming that the impact of common method bias in this study is not significant (Wen et al., 2018).

In summary, combining the results from the procedural controls and the three-stage *post hoc* statistical tests, it can be concluded that this study does not exhibit severe common method bias.

### Convergent validity and reliability analysis

The results of convergent validity and reliability analysis indicate that the measurement scales used are generally suitable for examining the theoretical model of this study. As shown in Table 3 (which presents standardized factor loadings, average variance extracted [AVE], and composite reliability [CR]), the measurement model demonstrates good psychometric properties: all constructs show satisfactory internal consistency, with their composite reliability (CR) values exceeding the critical standard of 0.70; the average variance extracted (AVE) values for most constructs also meet or approach the recommended threshold of 0.50.

**Table 3:** Factor loadings, reliability, and convergent validity.

Construct	Item	Standardized loading	AVE	CR
Low goal commitment	LGC1	0.78	0.60	0.88
	LGC2	0.79		
	LGC3	0.77		
	LGC4	0.71		
	LGC5	0.68		
Low self-transcendence	LST1	0.69	0.46	0.83
	LST2	0.69		
	LST3	0.66		
	LST4	0.63		
	LST5	0.68		
	LST6	0.65		
Low interpersonal communication	LIC1	0.77	0.53	0.85
	LIC2	0.77		
	LIC3	0.72		
	LIC4	0.68		
	LIC5	0.68		
High dependence	HD1	0.78	0.38	0.71
	HD2	0.69		
	HD3	0.45		
	HD4	0.54		
Family support	FamSup1	0.78	0.66	0.89
	FamSup2	0.79		
	FamSup3	0.80		
	FamSup4	0.75		
Friend Support	FriSup1	0.79	0.65	0.88
	FriSup2	0.77		
	FriSup3	0.76		
	FriSup4	0.74		
Other support	OthSup1	0.84	0.60	0.86
	OthSup2	0.72		
	OthSup3	0.50		
	OthSup4	0.41		
Social responsibility	SR1	0.67	0.500	0.923
	SR2	0.80		
	SR3	0.69		
	SR4	0.68		
	SR5	0.80		
	SR6	0.68		
	SR7	0.74		
	SR8	0.76		
	SR9	0.73		
	SR10	0.82		
	SR11	0.73		
	SR12	0.70		

It should be noted that the AVE value for the “High Dependence” dimension was 0.38. This finding is consistent with the original scale development study (Xu and Liu, 2022)—in the original study, this dimension also demonstrated the lowest reliability (CR = 0.702), and this value closely aligns with the CR value (0.71) for this construct in the present study, confirming the cross-sample stability of this dimension’s measurement properties. Additionally, the factor loading for item OthSup4 in the “Other Support” dimension was 0.41. Both of these items were retained for the following reasons: first, they hold significant theoretical importance for capturing the core connotation of their respective constructs; second, it is necessary to maintain consistency with the validated scales (Xu and Liu, 2022; Blumenthal et al., 1987). Sufficient evidence regarding discriminant validity and measurement invariance in subsequent sections further supports the robustness of this decision.

### Discriminant validity test

#### Confirmatory factor analysis and model fit

To test the discriminant validity among the eight variables in this study, Confirmatory Factor Analysis (CFA) was conducted using Amos 26.0 software on Low Goal Commitment, Low Self-Transcendence, Low Interpersonal Communication, High Dependence, Family Support, Friend Support, Other Support, and Social Responsibility. The results are shown in Table 4. The eight-factor model (*χ*^2^/df = 4.648, GFI = 0.842, RMSEA = 0.056, RMR = 0.047, CFI = 0.893, NFI = 0.867, NNFI = 0.884) had the best fit compared to the other seven alternative models, indicating that the discriminant validity among these eight variables measured by college students is good, and it is suitable for the subsequent hypothesis testing in this study.

**Table 4:** Confirmatory factor analysis results.

Factor model	*χ*^2^/*df*	GFI	RMSEA	RMR	CFI	NFI	NNFI
Eight-factor model	4.65	0.84	0.06	0.05	0.89	0.87	0.88
Seven-factor model	5.57	0.80	0.06	0.06	0.87	0.84	0.86
Six-factor model	6.64	0.77	0.07	0.06	0.83	0.81	0.82
Five-factor model	6.83	0.76	0.07	0.06	0.83	0.80	0.81
Four-factor model	10.37	0.66	0.09	0.11	0.72	0.70	0.70
Three-factor model	13.84	0.51	0.11	0.12	0.61	0.59	0.59
Two-factor model	14.95	0.42	0.11	0.14	0.58	0.56	0.56
One-factor model	16.74	0.40	0.12	0.14	0.52	0.51	0.50

#### HTMT criterion

To provide more sufficient evidence of discriminant validity, this study adopted the Heterotrait–Monotrait ratio of correlations (HTMT) as the testing criterion. Compared with traditional methods, this method has higher evaluation accuracy (Henseler et al., 2015). The results detailed in Table 5 show that most construct pairs have good discriminant validity.

**Table 5:** Heterotrait–Monotrait ratio (HTMT) of correlations.

Construct	1	2	3	4	5	6	7	8
1. Low goal commitment	–							
2. Low self-transcendence	0.69	–						
3. Low interpersonal communication	0.65	0.63	–					
4. High dependence	0.65	0.67	0.74	–				
5. Family support	0.32	0.15	0.30	0.19	–			
6. Friend support	0.24	0.11	0.33	0.23	0.77	–		
7. Other support	0.33	0.16	0.34	0.24	0.83	0.91	–	
8. Social responsibility	0.53	0.31	0.38	0.36	0.71	0.65	0.69	–

Among the four dimensions of “Buddha-like” Characteristics, the HTMT values between dimensions range from 0.63 to 0.74. This finding suggests that although these dimensions are conceptually components of the higher-order “Buddha-like” Characteristics construct, they demonstrate empirical distinctiveness. Crucially, the HTMT values between each dimension of “Buddha-like” Characteristics and the three dimensions of Social Support consistently remain at low levels (0.11–0.34), indicating a clear discriminant boundary between constructs from these two major theoretical domains. Social Responsibility shows moderate yet distinct associations with both the dimensions of “Buddha-like” Characteristics (HTMT values: 0.31–0.53) and the dimensions of Social Support (HTMT values: 0.65–0.71), further confirming its unique conceptual domain.

It should be noted that the HTMT value between Friend Support and Other Support is 0.911, slightly exceeding the conservative threshold of 0.85. This relatively high correlation may stem from contextual overlap within the Chinese university setting, where support provided by peers (Friend Support) and support from teachers or general classmates (Other Support) might be perceived with blurred boundaries by respondents. Importantly, this empirical finding is reasonably supported by the prior confirmatory factor analysis (CFA): the CFA results indicated that the hypothesized Eight-Factor Model (treating Friend Support and Other Support as distinct constructs) demonstrated a significantly better fit to the data compared to all alternative models (see Table 4). This consistent evidence from multiple analytical methods supports the rationale for treating them as independent constructs—ensuring theoretical precision while maintaining consistency with the original scale’s structure.

#### Measurement invariance

This study employed Mplus 8.3 software to examine measurement invariance across two grouping variables: School Category and Student Position. The results presented in Table 6 indicate that both groupings achieved the level of scalar invariance: all CFI differences (ΔCFI ≤ − 0.005) and RMSEA differences (ΔRMSEA ≤ |0.002|) met the stringent criteria of “ΔCFI ≤ 0.01 and ΔRMSEA ≤ 0.015,” indicating that the measurement model is equivalent across all subgroups.

**Table 6:** Measurement invariance test across groups.

Model	χ^2^	*df*	CFI	RMSEA	ΔCFI	ΔRMSEA
School category						
Configural invariance	1291.05	328	0.911	0.072	–	–
Metric invariance	1314.38	344	0.910	0.070	−0.001	−0.002
Scalar invariance	1372.93	360	0.906	0.070	−0.004	0.000
Student position						
Configural invariance	1297.83	328	0.907	0.072	–	–
Metric invariance	1326.26	344	0.906	0.071	−0.001	−0.001
Scalar invariance	1398.91	360	0.901	0.071	−0.005	0.000

This finding suggests that subsequent between-group comparisons are valid, and their results reflect true differences in the latent variables rather than measurement bias.

### Descriptive statistics, demographic variable differences, and correlation analysis

The means, standard deviations, “Buddha-like” Characteristics dimensions, and correlation coefficients are presented in Tables 7–9, respectively. In terms of descriptive statistics, the mean for Low Goal Commitment is 2.63 ± 0.85, the mean for Low Self-Transcendence is 3.05 ± 0.74, the mean for Low Interpersonal Communication is 2.84 ± 0.83, and the mean for High Dependence is 2.87 ± 0.68. The theoretical median of the “Buddha-like” Characteristics scale is 3, indicating that college students exhibit a moderate to low level of Low Goal Commitment, Low Interpersonal Communication, and High Dependence, while Low Self-Transcendence is at a moderate to high level. The mean for Family Support is 3.40 ± 0.8, the mean for Friend Support is 3.52 ± 0.72, and the mean for Other Support is 3.40 ± 0.74. The theoretical median for the Social Support scale is 3, indicating that college students show a moderate to high level of Social Support across all dimensions. The mean for social responsibility is 3.41 ± 0.6, and the theoretical median for this scale is 3, suggesting that college students have a moderate to high level of Social Responsibility.

**Table 7:** Descriptive statistics of key variables (*n* = 1,147).

Variable	Min	Max	M	SD
Low goal commitment	1.00	5.00	2.63	0.85
Low self-transcendence	1.00	5.00	3.05	0.74
Low interpersonal communication	1.00	5.00	2.84	0.83
High dependence	1.00	5.00	2.87	0.68
Family support	1.00	5.00	3.40	0.80
Friend support	1.00	5.00	3.52	0.72
Other support	1.00	5.00	3.40	0.74
Social responsibility	1.00	5.00	3.41	0.60

**Table 8:** Demographic differences in “Buddha-like” dimensions (*n* = 1,147).

Variable	Low goal commitment	Low self-transcendence	Low interpersonal communication	High dependence
Gender	−0.75	−1.30	0.70	−0.46
Grade	1.80	1.18	4.14^**^	1.18
School category	2.83^**^	0.95	3.52^***^	0.11
Major	2.71^*^	2.41^*^	1.88	0.93
Student position	−4.46^***^	−6.07^***^	−8.25^***^	−3.78^***^
Scholarship status	−4.31^***^	−3.66^***^	−2.57^*^	−2.78^**^
Honor status	−4.34^***^	−4.46^***^	−3.96^***^	−2.59^*^
Place of origin	0.37	0.30	0.41	0.07

^***^p < 0.001, ^**^p < 0.01, ^*^p < 0.05.

**Table 9:** Correlation analysis of key variables (*n* = 1,147).

Variable	1	2	3	4	5	6	7	8	9	10	11
Student position	1										
Scholarship status	0.21^**^	1									
Honor status	0.31^**^	0.50^**^	1								
Low goal commitment	0.13^**^	0.13^**^	0.13^**^	1							
Low Self-transcendence	0.18^**^	0.11^**^	0.13^**^	0.59^**^	1						
Low interpersonal communication	0.24^**^	0.08^**^	0.12^**^	0.56^**^	0.53^**^	1					
High dependence	0.11^**^	0.08^**^	0.08^**^	0.51^**^	0.52^**^	0.57^**^	1				
Family support	−0.09^**^	−0.09^**^	−0.09^**^	−0.28^**^	−0.10^**^	−0.26^**^	−0.11^**^	1			
Friend support	−0.08^**^	−0.04	−0.10^**^	−0.21^**^	−0.03	−0.29^**^	−0.14^**^	0.68^**^	1		
Other support	−0.09^**^	−0.09^**^	−0.12^**^	−0.28^**^	−0.10^**^	−0.29^**^	−0.15^**^	0.73^**^	0.79^**^	1	
Social responsibility	−0.13^**^	−0.15^**^	−0.17^**^	−0.48^**^	−0.25^**^	−0.34^**^	−0.27^**^	0.64^**^	0.58^**^	0.61^**^	1

^**^p < 0.01, ^*^p < 0.05.

Regarding the analysis of differences in demographic characteristic variables, independent samples *t*-tests and one-way ANOVAs were used based on the corresponding sample sizes for the demographic characteristic variables. Specifically, independent samples *t*-tests were applied to Gender, School category, Student position, Scholarship status, and honor status; one-way ANOVAs were applied to Grade, Major, and Place of Origin. The results show that, in terms of Grade, sophomore and junior students had significantly higher scores in Low Interpersonal Communication than freshman students. In terms of School category, undergraduate students had significantly higher scores in Low Goal Commitment and Low Interpersonal Communication compared to vocational college students. Regarding the Major, students in the humanities, science, and agriculture had significantly higher scores in Low Goal Commitment and Low Self-Transcendence compared to those in business and economics, engineering, and other majors. Additionally, college students who did not hold any positions, had not received scholarships, or had not been awarded Honors scored significantly higher in all dimensions of “Buddha-like” Characteristics compared to those who held positions or had received Scholarships and Honors. No significant differences were found based on Gender or Place of Origin.

In the correlation analysis, among the control variables, only Student Position, Scholarship Status, and Honor Status showed significant correlations with the independent and dependent variables (except for the Scholarship Status, which did not show a significant correlation with friend support). Low Goal Commitment was significantly negatively correlated with Family Support, Friend Support, and Other Support (*r* = −0.28, *p* < 0.01; *r* = −0.21, *p* < 0.01; *r* = −0.28, *p* < 0.01), and also with social responsibility (*r* = −0.48, *p* < 0.01). Low Self-Transcendence was significantly negatively correlated with Family Support and other support (*r* = −0.10, *p* < 0.01; *r* = −0.1, *p* < 0.01), and also with social responsibility (*r* = −0.25, *p* < 0.01). Low Interpersonal Communication was significantly negatively correlated with Family Support, Friend Support, and Other Support (*r* = −0.26, *p* < 0.01; *r* = −0.29, *p* < 0.01; *r* = −0.29, *p* < 0.01), and also with Social Responsibility (*r* = −0.34, *p* < 0.01). High Dependence was significantly negatively correlated with Family Support, Friend Support, and Other Support (*r* = −0.11, *p* < 0.01; *r* = −0.14, *p* < 0.01; *r* = −0.15, *p* < 0.01), and also with Social Responsibility (*r* = −0.27, *p* < 0.01). These results suggest that the preliminary analysis is generally consistent with the hypotheses. Furthermore, the correlation coefficients between the corresponding variables are all below 0.7, which preliminarily indicates that there is no potential multicollinearity issue among the variables involved.

### Hypothesis testing

#### Mechanism Model of the Role of Social Support in “Buddha-like” Characteristics and Social Responsibility

To examine the overall mediating role of Social Support in the relationship between “Buddha-like” Characteristics and Social Responsibility, this study utilized Amos 26.0 to construct a two-second-order structural equation model that included control variables. This model aggregated the four first-order dimensions—“Low Goal Commitment,” “Low Self-Transcendence,” “Low Interpersonal Communication,” and “High Dependence”—into the second-order factor of “Buddha-like” characteristics. Simultaneously, it aggregated “Family Support,” “Friend Support,” and “Other Support” into the second-order factor of Social Support. “Student Position,” “Scholarship Status,” and “Honor Status” were included as control variables.

#### Model fit evaluation

The model goodness-of-fit indices indicated that the theoretical model demonstrated an generally acceptable fit to the data. The specific fit indices were as follows: *χ*^2^/df = 4.954, CFI = 0.867, TLI = 0.859, RMSEA = 0.059, SRMR = 0.055 (see Table 10). Although the CFI and TLI values were slightly below the excellent standard of 0.90, considering the model complexity and sample size, the RMSEA and SRMR indices performed satisfactorily, suggesting that the model is acceptable.

**Table 10:** Structural equation model fit indices (*n* = 1,147).

	χ^2^/*df*	CFI	TLI	RMSEA	SRMR	GFI
Model	4.954	0.867	0.859	0.059	0.055	0.821

#### Reliability and validity of the measurement model

**Paths between second-order and first-order latent variables.** Analysis of the second-order factor paths provided support for the theoretical construct structure of this study. As shown in Table 11, the standardized loadings between the second-order factor of “Buddha-like” Characteristics and its first-order dimensions (Low Goal Commitment, Low Self-Transcendence, Low Interpersonal Communication, and High Dependence) were highly significant (range: 0.797–0.832). Similarly, the second-order factor of Social Support also demonstrated highly significant and strong associations with its first-order dimensions (Family Support, Friend Support, and Other Support), with loadings ranging from 0.855 to 0.982. These results indicate that each first-order dimension effectively converges into its corresponding theoretical higher-order factor, empirically supporting the rationality of modeling “Buddha-like” Characteristics and Social Support as multidimensional constructs.

**Table 11:** Path indices of second-order latent and first-order latent variables (*n* = 1,147).

Second-order latent variables	First-order latent variables	Standardized loadings
“Buddha-like” characteristics	Low goal commitment	0.832
	Low self-transcendence	0.805
	Low interpersonal communication	0.797
	High dependence	0.833
Social support	Family support	0.855
	Friend support	0.913
	Other support	0.982

**Paths between first-order latent variables and items.** All measurement items demonstrated statistically significant factor loadings on their corresponding first-order latent variables (*p* < 0.001). The standardized factor loadings for the vast majority of items exceeded the acceptable threshold of 0.60. Two items exhibited slightly lower loadings but were retained due to their theoretical importance:

The LST3 item under the Low Self-Transcendence dimension (“I prefer a comfortable and stable life”) showed a standardized factor loading of 0.489. This item directly reflects the core concept of “preference for stability and avoidance of challenges” and was therefore retained. The dimension demonstrated satisfactory convergent validity overall, with a reasonably high loading (0.805) on the second-order “Buddha-like” Characteristics factor, indicating that the lower loading of this individual item did not substantially compromise the construct validity of the dimension.

The HD1 item under the High Dependence dimension (“I rarely refuse when others assign tasks to me”) showed a standardized factor loading of 0.434. This item was fixed as a reference indicator (with its unstandardized coefficient fixed at 1.000) and represents typical behavior reflecting “lack of assertiveness and difficulty in refusal,” which is crucial for the content validity of the construct. Consequently, it was retained, and the overall validity of this dimension within the model remained unaffected.

#### Direct effect test

**Figure 1:**
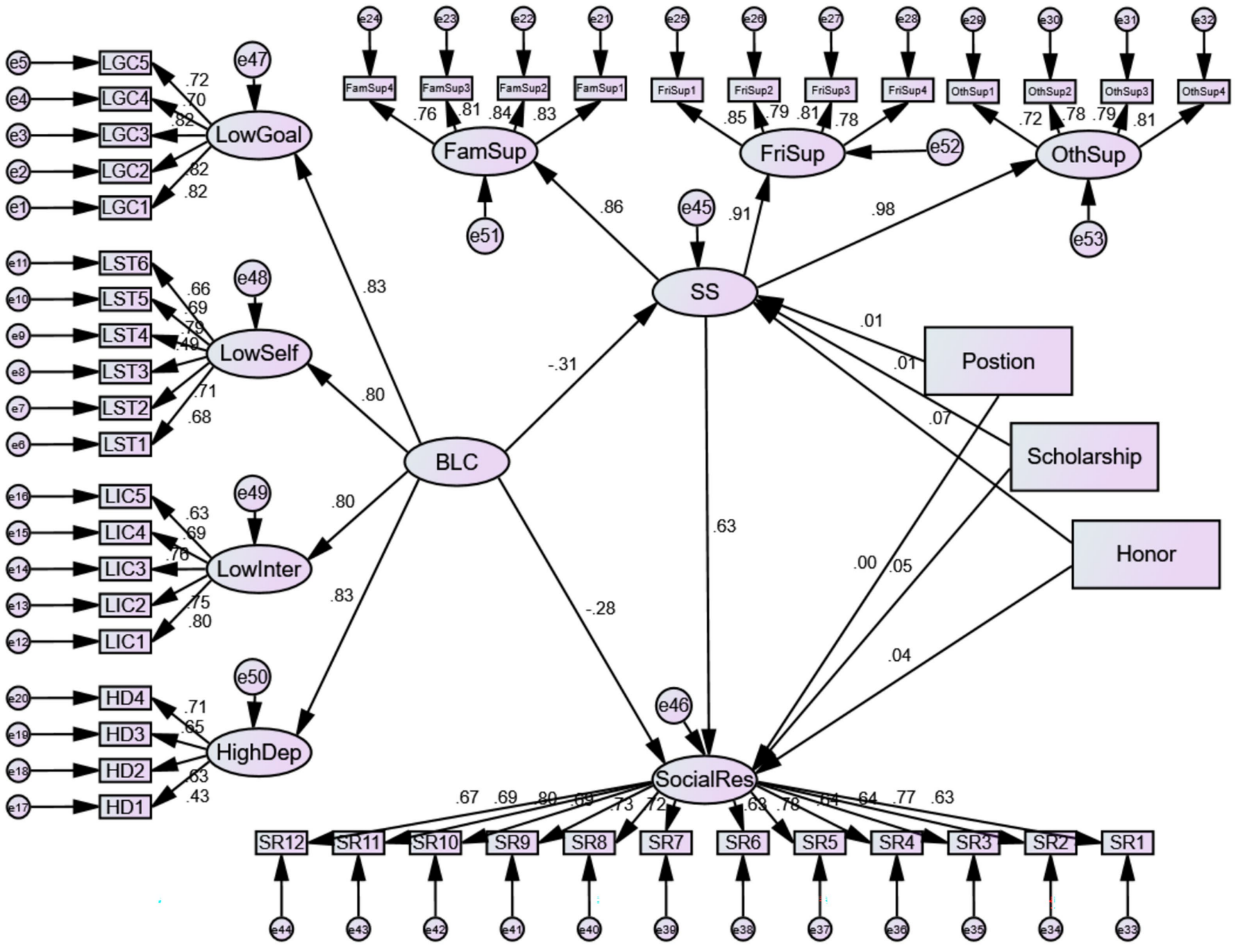
Structural equation model of “Buddha-like” characteristics, social support, and social responsibility.

After controlling for Student Position, Scholarship Status, and Honor Status, the path coefficients of the structural equation model and their Bootstrap confidence intervals (with 5,000 resamples) clearly revealed the direct relationships between variables (standardized path coefficients are shown in Figure 1).

A significant negative direct relationship was observed between “Buddha-like” Characteristics and Social Responsibility (unstandardized estimate = −0.230, S. E. = 0.023, C. R. = − 9.906, *p* < 0.001; standardized estimate *β* = −0.285), supporting Hypothesis H1.

A significant negative relationship was found between “Buddha-like” Characteristics and Social Support (unstandardized estimate = −0.287, S. E. = 0.033, C. R. = − 8.729, *p* < 0.001; standardized estimate *β* = −0.307), supporting Hypothesis H2.

A significant positive relationship was demonstrated between Social Support and Social Responsibility (unstandardized estimate = 0.546, S. E. = 0.033, C. R. = 16.699, *p* < 0.001; standardized estimate *β* = 0.633), supporting Hypothesis H3.

To examine the mediating role of Social Support, this study employed the Bootstrap method, conducting 5,000 repeated samplings on the 1,147 samples to calculate the 95% confidence interval for the mediating effect. The results (see Table 12) showed that the total relationship between “Buddha-like” Characteristics and Social Responsibility was −0.387 (95% CI [−0.459, −0.319]). The indirect relationship through Social Support was −0.157, with its 95% confidence interval [−0.209, −0.113] excluding zero, indicating that this mediating relationship is statistically significant. After controlling for the mediating pathway, the direct relationship between “Buddha-like” Characteristics and Social Responsibility remained significant (effect value = −0.230, 95% CI [−0.289, −0.175]).

**Table 12:** Results of mediating effect analysis.

Effect type	Estimate	SE	95% Bootstrap confidence interval		*p*-value
			Lower bound	Upper bound	
Indirect effect	−0.157	0.024	−0.209	−0.113	<0.001
Direct effect	−0.230	0.029	−0.289	−0.175	<0.001
Total effect	−0.387	0.036	−0.459	−0.319	<0.001

Since both the direct and indirect relationships were significant, this study concludes that Social Support plays a significant partial mediating role between “Buddha-like” Characteristics and Social Responsibility. This mediating relationship accounts for 40.6% of the total relationship. In summary, research hypothesis H4 is supported by the data.

#### Robustness test of the model

To evaluate the robustness of the key findings, this study conducted a sensitivity analysis by comparing structural equation models with and without control variables. The results showed that the key parameters of the model remained stable.

**Path coefficients.** The changes in standardized coefficients for the core paths were minimal (*Δ*β ≤ 0.016). The coefficient for the path from “Buddha-like” Characteristics to Social Support changed from −0.321 to −0.307, the coefficient from Social Support to Social Responsibility remained stable at 0.633, and the direct path coefficient from “Buddha-like” Characteristics to Social Responsibility changed from −0.293 to −0.285.

**Mediating relationship.** The proportion of the mediating relationship of Social Support to the total relationship slightly changed from 40.9 to 40.6% (Δ = 0.3%). The Bootstrap test (5,000 resamples) reconfirmed a significant indirect relationship (effect value = −0.157, 95% CI [−0.209, −0.113]), with the bias close to zero (Mean Bias = 0.000).

**Model fit.** Changes in the overall model fit indices were within acceptable ranges (RMSEA: 0.058 → 0.059; CFI: 0.885 → 0.867).

In summary, after incorporating the control variables, the core paths, mediating mechanism, and overall structure of the theoretical model in this study did not undergo substantial changes. This indicates that the partial mediating role of Social Support is a robust finding.

#### Dimensional heterogeneity analysis based on hierarchical regression

Although the structural equation model validated the theoretical relationships at the overall level, it could not fully reveal the complex mechanisms among the sub-dimensions. To further investigate the unique net effects of each dimension of “Buddha-like” Characteristics on Social Responsibility after controlling for each other, and to dissect the specific mediating pathways of each dimension of Social Support, this study employed hierarchical regression analysis for supplementary testing, utilizing the Bootstrap method for verification.

#### Effects of dimensions of “Buddha-like” characteristics and social support on social responsibility

Using Social Responsibility as the dependent variable, the four dimensions of “Buddha-like” characteristics (Low Goal Commitment, Low Self-Transcendence, Low Interpersonal Communication, High Dependence) as independent variables (mean-centered), the three dimensions of Social Support (Family Support, Friend Support, Other Support) as mediating variables (mean-centered), and Student Position, Scholarship Status, and Honor Status as control variables (dummy-coded), multiple linear regression was performed using the hierarchical regression method.

The analysis results (Table 13) clearly revealed the action paths among variables. First, in Model 1, when only the dimensions of “Buddha-like” characteristics were included, Low Goal Commitment (*β* = −0.446, *p* < 0.001) and Low Interpersonal Communication (*β* = −0.121, *p* < 0.001) showed significant direct negative associations with Social Responsibility, whereas Low Self-Transcendence showed a significant positive association (*β* = 0.103, *p* < 0.01), preliminarily revealing the complexity of its internal functioning. H1a and H1c were supported. This model accounted for 25.5% of the variance in Social Responsibility (*R*^2^ = 0.255), with a large effect size (*f*^2^ = 0.289).

**Table 13:** Regression analysis of the relationships among dimensions of “Buddha-like” characteristics, dimensions of social support, and social responsibility (*n* = 1,147).

Variables	Model 1: social responsibility β (95%CI)	Model 2: family support β (95%CI)	Model 3: friend support β (95%CI)	Model 4: other support β (95%CI)	Model 5: social responsibility β (95%CI)
Control variables					
Student position	0.026 (−0.034 ~ 0.097)	0.015 (−0.070 ~ 0.118)	0.002 (−0.082 ~ 0.088)	0.008 (−0.075 ~ 0.097)	0.020 (−0.027 ~ 0.074)
Scholarship Status	0.049 (−0.011 ~ 0.135)	0.040 (−0.039 ~ 0.171)	−0.017 (−0.121 ~ 0.069)	0.023 (−0.061 ~ 0.132)	0.036 (−0.011 ~ 0.102)
Honor Status	0.078^*^ (0.021 ~ 0.166)	0.034 (−0.049 ~ 0.159)	0.082^*^ (0.025 ~ 0.213)	0.069^*^ (0.007 ~ 0.198)	0.041 (−0.006 ~ 0.106)
“Buddha-like” characteristics					
Low goal commitment	−0.446^***^ (−0.363 ~ −0.267)	−0.276^***^ (−0.328 ~ −0.189)	−0.174^***^ (−0.210 ~ −0.085)	−0.253^***^ (−0.282 ~ −0.155)	−0.288^***^ (−0.242 ~ −0.165)
Low self-transcendence	0.103^**^ (0.030 ~ 0.139)	0.149^***^ (0.082 ~ 0.239)	0.238^***^ (0.162 ~ 0.304)	0.172^***^ (0.100 ~ 0.244)	−0.014 (−0.055 ~ 0.031)
Low interpersonal communication	−0.121^***^ (−0.139 ~ −0.039)	−0.226^***^ (−0.290 ~ −0.147)	−0.308^***^ (−0.334 ~ −0.204)	−0.258^***^ (−0.296 ~ −0.164)	0.046 (−0.006 ~ 0.073)
High dependence	−0.009 (−0.067 ~ 0.050)	0.089^*^ (0.022 ~ 0.190)	0.006 (−0.069 ~ 0.082)	0.050 (−0.022 ~ 0.131)	−0.047 (−0.087 ~ 0.003)
Social support					
Family support					0.334^***^ (0.208 ~ 0.296)
Friend support					0.191^***^ (0.103 ~ 0.215)
Other support					0.130^***^ (0.048 ~ 0.164)
Model fit indices					
*R* ^2^	0.255	0.120	0.123	0.134	0.556
Adjusted *R*^2^	0.250	0.115	0.118	0.128	0.552
Δ*R*^2^	0.215	0.105	0.111	0.115	0.301
*F*-value	55.689	22.202	22.901	25.117	142.434
*f*^2^effect size	0.289	0.119	0.125	0.134	0.678

^*^p < 0.05, ^**^p < 0.01, ^***^p < 0.001.

Models 2 to 4 tested the effects of each dimension of “Buddha-like” characteristics on the three dimensions of Social Support, respectively. Specifically, Low Goal Commitment showed significant negative associations with all three dimensions of Social Support (Family Support: *β* = −0.276, *p* < 0.001; Friend Support: *β* = −0.174, *p* < 0.001; Other Support: *β* = −0.253, *p* < 0.001), supporting H2a, H2b, and H2c. Low Interpersonal Communication also showed significant negative associations with all three dimensions of Social Support (Family Support: *β* = −0.226, *p* < 0.001; Friend Support: *β* = −0.308, *p* < 0.001; Other Support: *β* = −0.258, *p* < 0.001), with the strongest negative association observed for Friend Support, supporting H2g, H2h, and H2i. Notably, Low Self-Transcendence showed stable positive associations with all dimensions of Social Support (Family Support: *β* = 0.149, *p* < 0.001; Friend Support: *β* = 0.238, *p* < 0.001; Other Support: *β* = 0.172, *p* < 0.001), and High Dependence also showed a significant positive association with Family Support (*β* = 0.089, *p* < 0.05), a direction opposite to that of the other dimensions.

In the full Model 5, which included the Social Support variables, all three dimensions of Social Support demonstrated significant positive associations with Social Responsibility. Among them, Family Support showed the strongest association (*β* = 0.334, *p* < 0.001), followed by Friend Support (*β* = 0.191, *p* < 0.001) and Other Support (*β* = 0.130, *p* < 0.001). The overall explanatory power of this model improved significantly, with *R*^2^ reaching 0.556, an increase of 0.301 compared to Model 1’s *R*^2^, and an extremely large effect size (*f*^2^ = 0.678), indicating that the inclusion of Social Support substantially enhanced the ability to account for variance in Social Responsibility. Crucially, compared to Model 1 containing only “Buddha-like” characteristics, after controlling for Social Support, the direct effects of Low Self-Transcendence and Low Interpersonal Communication on Social Responsibility became non-significant, suggesting that their influence on Social Responsibility is fully mediated by Social Support. In contrast, Low Goal Commitment maintained a significant direct negative association (*β* = −0.288, *p* < 0.001), indicating partial mediation. Furthermore, the variance inflation factor (VIF) for all variables in the model was well below 10, indicating that the analysis results were not confounded by multicollinearity issues (Hair et al., 1995).

The analysis of the net effects of the “Buddha-like” dimensions revealed a complex preliminary pattern. Contrary to the results of the bivariate correlation analysis (*r* = −0.25, *p* < 0.01) (see Table 9), in the hierarchical regression Model 1, after controlling for the other “Buddha-like” dimensions, the standardized coefficient of Low Self-Transcendence on Social Responsibility reversed to a significant positive association (*β* = 0.103, *p* < 0.01). This phenomenon suggests that within the “Buddha-like” Characteristics, dimensions such as Low Goal Commitment might exert a statistical suppression effect on the relationship between Low Self-Transcendence and Social Responsibility, potentially masking the latter’s unique predictive pattern.

#### Mediating effect analysis

To systematically examine the mediating mechanism of Social Support between “Buddha-like” Characteristics and Social Responsibility, this study utilized the SPSSAU online statistical analysis platform to construct a parallel multiple mediation model. The model specified Family Support, Friend Support, and Other Support as mediating variables; Low Goal Commitment, Low Self-Transcendence, Low Interpersonal Communication, and High Dependence as independent variables; and Social Responsibility as the dependent variable. Under the condition of controlling for Student Position, Scholarship Status, and Honor Status, Bootstrap sampling with 5,000 iterations was employed to calculate 95% confidence intervals, testing the significance of each mediating pathway. This analysis strictly followed the mediation testing framework proposed by Hayes (2013), whose statistical logic is compatible with SPSS’s PROCESS macro, ensuring the scientific validity and reliability of the analytical results. The specific analysis results are presented in Table 14.

**Table 14:** Results of mediating effect test (Bootstrap = 5,000).

Paths	Total effect	Path a	Path b	Mediating effect	95% Boot CI	Direct effect	Proportion of effect	Conclusion
LowGoal → FamSup → SocialRes	−0.315^**^	−0.258^**^	0.252^**^	−0.065	−0.126 ~ −0.060	−0.203^**^	20.66%	Partial mediation
LowGoal → FriSup → SocialRes	−0.315^**^	−0.147^**^	0.159^**^	−0.023	−0.056 ~ −0.015	−0.203^**^	7.44%	Partial mediation
LowGoal → OthSup → SocialRes	−0.315^**^	−0.219^**^	0.106^**^	−0.023	−0.061 ~ −0.009	−0.203^**^	7.36%	Partial mediation
LowSelf → FamSup → SocialRes	0.084^**^	0.161^**^	0.252^**^	0.041	0.024 ~ 0.077	−0.012	100%	Full mediation
LowSelf → FriSup → SocialRes	0.084^**^	0.233^**^	0.159^**^	0.037	0.024 ~ 0.071	−0.012	100%	Full mediation
LowSelf → OthSup → SocialRes	0.084^**^	0.172^**^	0.106^**^	0.018	0.006 ~ 0.043	−0.012	100%	Full mediation
LowInter → FamSup → SocialRes	−0.089^**^	−0.219^**^	0.252^**^	−0.055	−0.109 ~ −0.046	0.034	100%	Full mediation
LowInter → FriSup → SocialRes	−0.089^**^	−0.269^**^	0.159^**^	−0.043	−0.091 ~ −0.031	0.034	100%	Full mediation
LowInter → OthSup → SocialRes	−0.089^**^	−0.230^**^	0.106^**^	−0.024	−0.060 ~ −0.010	0.034	100%	Full mediation
HighDep → FamSup → SocialRes	−0.008	0.106*	0.252^**^	0.027	0.001 ~ 0.061	−0.042	100%	Full mediation
HighDep → FriSup → SocialRes	−0.008	0.006	0.159^**^	0.001	−0.018 ~ 0.018	−0.042	0%	Mediating effect not significant
HighDep → OthSup → SocialRes	−0.008	0.055	0.106^**^	0.006	−0.004 ~ 0.022	−0.042	0%	Mediating effect not significant

^*^p < 0.05, ^**^p < 0.01.

The Bootstrap mediation analysis results revealed differentiated mediating mechanisms of the various dimensions of Social Support between the dimensions of “Buddha-like” Characteristics and Social Responsibility.

First, the mediating pathways of Low Goal Commitment through all three dimensions of Social Support were significant, with a total indirect effect of −0.112 (95% CI [−0.206, −0.111]), indicating partial mediation. Therefore, hypotheses H4a, H4b, and H4c are supported. Among these, the mediating effect through Family Support was the strongest (effect size = −0.065), suggesting that the negative relationship between Low Goal Commitment and Social Responsibility is primarily transmitted through the core pathway of suppressing Family Support.

Second, both Low Self-Transcendence and Low Interpersonal Communication achieved full mediation through all three dimensions of Social Support, supporting hypotheses H4d, H4e, H4f, H4g, H4h, and H4i. Specifically: Low Self-Transcendence generated positive indirect effects through all three Social Support dimensions (total indirect effect = 0.096), with the largest effect occurring through Family Support (effect size = 0.041). Low Interpersonal Communication showed the strongest negative association with Friend Support (path a = −0.269) and produced a negative indirect effect through Social Support (total indirect effect = −0.122). Notably, the pathways through Other Support for both dimensions also reached significance (Low Self-Transcendence: 0.018, 95% CI [0.006, 0.043]; Low Interpersonal Communication: −0.024, 95% CI [−0.060, −0.010]), indicating that social support from sources like teachers and classmates, beyond family and friends, also serves as an important channel transmitting the influence of “Buddha-like” Characteristics.

Finally, the total effect of High Dependence on Social Responsibility was not significant (effect size = −0.008, *p* = 0.183). However, its mediating effect through Family Support reached significance (effect size = 0.027, 95% CI = [0.001, 0.061]), thus supporting H4j. In contrast, the mediating effects through Friend Support and Other Support were not significant, so H4k and H4l are not supported.

The Bootstrap-based mediation test not only verified the mediating role of Social Support but also identified two distinct types of mediation models. The relationship between High Dependence and Social Responsibility exhibited a typical inconsistent mediation pattern. Its non-significant total effect (effect size = −0.008) was not due to the dimension being ineffective, but rather resulted from the competition and offset between its significant positive indirect effect through Family Support (effect size = 0.027) and its negative direct effect (effect size = −0.042). This suggests that High Dependence might involve dual mechanisms: it indirectly promotes Social Responsibility by enhancing the sense of support in family contexts, but this positive pathway is counteracted by other unmeasured negative effects, ultimately leading to a non-significant total effect.

Simultaneously, Social Support played a dual role as both a suppressor variable and a mediating variable in the relationship between Low Self-Transcendence and Social Responsibility. After controlling for Social Support, the direct effect of Low Self-Transcendence became non-significant and its direction reversed to negative (effect size = −0.012). According to the criteria defined by MacKinnon et al. (2000), this pattern—where the inclusion of the mediator leads to substantial changes in the sign or significance of the total and direct effects of the independent variable on the dependent variable—constitutes clear evidence of a statistical suppression effect (MacKinnon et al., 2000). This suppression effect reveals the complexity of Low Self-Transcendence’s role: its superficially weak “positive” total effect (effect size = 0.084) is a statistical artifact. Path analysis clarifies the source of this artifact: Low Self-Transcendence influences Social Responsibility through two opposing pathways:

**Positive indirect pathway.** It showed significant positive associations with Family Support (path a = 0.161), Friend Support (path a = 0.233), and Other Support (path a = 0.172). This association is more likely derived from a form of “passive support dependence”—where individuals passively maintain a low-investment, superficial social network to avoid challenges and risks.

**Negative direct pathway.** After excluding the mediating influence of Social Support, its net direct effect was negative (effect size = −0.012). Although not statistically significant, it still hints at an underlying negative tendency.

Ultimately, the positive indirect effect (effect size = 0.096) and the negative direct effect (effect size = −0.012) offset each other, resulting in a weakly positive total effect. The Bootstrap test confirmed that Social Support plays a full mediating role in this context.

## Discussion

Grounded in social cognitive theory and integrating structural equation modeling with multiple statistical methods, this study systematically examined the relationship between “Buddha-like” characteristics and Social Responsibility among college students. The main findings are as follows:

### Validation of the overall model: “Buddha-like” characteristics as a higher-order construct and its dual pathways

The two-second-order structural equation model constructed in this study demonstrated acceptable fit indices (*χ*^2^/df = 4.954, CFI = 0.867, TLI = 0.859, RMSEA = 0.059, SRMR = 0.055), indicating reasonable model adequacy. This finding not only validates the localized structure of “Buddha-like” Characteristics but also provides important cross-cultural evidence for understanding youth mentalities and behavioral patterns globally.

#### The four-dimensional structure of “Buddha-like” characteristics and cross-cultural comparison

The model aggregated the four dimensions—“Low Goal Commitment, Low Self-Transcendence, Low Interpersonal Communication, and High Dependence”—into the higher-order factor of “Buddha-like” characteristics. The standardized factor loadings of the first-order dimensions ranged from 0.797 to 0.832, and both reliability and validity indices met psychometric standards. This result empirically supports the four-dimensional structure theory of “Buddha-like” characteristics proposed by Xu and Liu (2022), indicating that “Buddha-like” characteristics constitute an internally consistent multidimensional construct. Its relationship with Social Responsibility is systematic and holistic, rather than a simple additive effect of isolated traits.

From a cross-cultural perspective, “Buddha-like” characteristics share commonalities with phenomena like “quiet quitting” in Western societies, yet also exhibit profound differences. The commonality lies in both potentially stemming from individuals’ motivation regulation and behavioral withdrawal in the face of social pressure. However, their domains of manifestation and core logic are distinct: “Quiet quitting” in Europe and America is primarily defined as a boundary management strategy confined to the workplace, where employees strictly limit their efforts to job descriptions to cope with job dissatisfaction and pursue work-life balance (Mahand and Caldwell, 2023; Bernuzzi et al., 2025). In contrast, the “Buddha-like” mindset among Chinese college students manifests as a systematic psychological withdrawal permeating multiple life domains. This difference profoundly reflects culture’s role in shaping self-construal (Markus and Kitayama, 1991): in individualistic cultures, the self is independent and bounded, and youth maintain their core independence by setting clear boundaries in specific areas (e.g., work); whereas in China’s collectivist culture, the self is interdependent and more flexible, leading youth to adopt a dynamic, all-encompassing psychological and behavioral adjustment to comply with external demands and maintain relational harmony.

#### Dual pathways linking “Buddha-like” characteristics to social responsibility: universal mechanisms and cultural specificity

Structural equation modeling analysis revealed that “Buddha-like” characteristics relate to Social Responsibility through dual pathways:

**Direct negative pathway.** After controlling for Social Support and demographic variables, a significant direct negative relationship was observed between “Buddha-like” characteristics and Social Responsibility (*β* = −0.285, *p* < 0.001). This suggests that these characteristics themselves may directly weaken individuals’ sense of responsibility and related behavioral tendencies, consistent with Tong’s (2023) conclusion regarding the association between “Buddha-like” traits and diminished social responsibility. This pathway can be explained from a motivational perspective: the Low Goal Commitment within “Buddha-like” characteristics shares behavioral similarities with the state of amotivation defined by Self-Determination Theory (Ryan and Deci, 2000), both exhibiting insufficient goal-driven motivation.

**Indirect negative pathway.** The indirect relationship of “Buddha-like” characteristics through Social Support was also significant, accounting for 40.6% of the total relationship. This finding aligns with the “individual-environment” interaction mechanism in social cognitive theory (Bandura, 1986): “Buddha-like” characteristics not only directly relate to responsibility awareness (an individual factor) but also may indirectly hinder responsible behavior by reducing access to Social Support (an environmental factor). Together, these pathways form a comprehensive mechanism linking “Buddha-like” characteristics to Social Responsibility.

The dual pathways through which “Buddha-like” Characteristics influence Social Responsibility provide a culturally contextualized empirical case for the universality of social cognitive theory. However, the strong mediating role of Social Support within the indirect pathway highlights the specificity of the Chinese context: in collectivist cultures emphasizing interpersonal connections, environmental factors exert a particularly prominent influence on individual responsibility development. This stands in sharp contrast to responsibility fulfillment patterns in Western individualistic cultures, which place greater emphasis on individual autonomy and intrinsic value drive (Chirkov et al., 2003).

### The key mediating role of social support: family support as the Core protective mechanism

On the basis of confirming the mediating role of social support, the Bootstrap multiple mediating effect test further revealed the functional heterogeneity among its internal dimensions and highlighted the core position of family support within this mechanism.

#### The overall buffering effect of social support

The mediating effect of Social Support accounted for 40.6% of the total relationship, indicating that nearly half of the negative association between “Buddha-like” characteristics and Social Responsibility was transmitted through the pathway of “inhibiting the acquisition of Social Support.” This result strongly supports the “social support buffering hypothesis” proposed by Cohen and Wills (1985), suggesting that among college students, Social Support can serve as an environmental resource, potentially mitigating the lack of motivation and cognitive biases associated with “Buddha-like” characteristics. For instance, individuals with Low Goal Commitment might receive value guidance through Family Support, while those with Low Interpersonal Communication could rebuild social connections through Friend Support, thereby indirectly fostering the development of responsibility awareness.

#### The dominant role of family support

Significant differences existed in the mediating effects across the dimensions of Social Support, with Family Support demonstrating the strongest effect across all pathways. Taking Low Goal Commitment as an example, its mediating effect through Family Support was −0.065 (95% CI = [−0.126, −0.060]), accounting for 58.56% of the total mediating effect for this dimension, underscoring the central role of Family Support in explaining the relationship involving Low Goal Commitment. Concurrently, in the hierarchical regression model (Model 5), Family Support also showed the strongest direct positive association with Social Responsibility (*β* = 0.334, *p* < 0.001). This aligns with the view put forward by Shen and Li (2020): within the Chinese cultural context, the family, as the earliest and most stable source of support for individuals, plays a far greater role in shaping responsibility cognition through emotional nourishment and value transmission than other social relationships. The mediating effects of Friend Support and Other Support were relatively weaker; for example, the mediating effect of Low Goal Commitment through Friend Support was −0.023 (accounting for 20.72%). This disparity reflects the “match between support functions and individual needs”: the “goal deficiency” in “Buddha-like” characteristics requires more of the “value guidance” from family (family function), while the “social interaction deficiency” requires the “interactive connection” from friends (friend function). In contrast, “Other Support” from teachers or ordinary classmates, limited by its emotional depth, offers a relatively weaker buffering effect, further refining the theory of functional specificity regarding social support.

The finding that Family Support exhibited the strongest effect across all pathways is not a coincidence but an inevitable outcome deeply rooted in cultural logic. In collectivist cultures like China, the self is constructed as an interdependent “relational self” (Markus and Kitayama, 1991), where an individual’s identity and sense of responsibility are highly dependent on close family ties. Therefore, family support serves not only as emotional solace but also as the primary vehicle for value inculcation, goal setting, and role norm transmission.

The dominant role of Family Support highlights the cultural divergence in the functions of support systems: in individualistic cultures, individual psychological adaptation relies more on horizontally chosen support sources like peer networks; whereas the responsibility formation of Chinese youth depends more on the vertical, stable support source of the family for value guidance and responsibility transmission (Triandis, 2001). Consequently, the relatively weaker effects of Friend Support and Other Support in this study precisely reflect the “family-centric” nature of support functions within the Chinese cultural context, providing key evidence for understanding how culture shapes developmental resources.

### Differentiated impacts of “Buddha-like” characteristics: dimensional heterogeneity and group differences

Hierarchical regression analysis revealed the distinct mechanisms through which the four dimensions of “Buddha-like” characteristics relate to Social Responsibility, while demographic difference analysis supplemented the understanding of variations in these characteristics from a group perspective.

#### Heterogeneous mechanisms across dimensions: from “primary direct contributor” to “full mediation”

Low Goal Commitment emerged as the primary direct contributor to reduced Social Responsibility. In the model including only the “Buddha-like” dimensions, it demonstrated the strongest negative association (*β* = −0.446, *p* < 0.001), which remained significant even after controlling for Social Support (*β* = −0.288, *p* < 0.001). This suggests that “goal deficiency” directly weakens motivation for responsibility and is difficult to be fully compensated by Social Support (Locke and Latham, 1990).

Both Low Self-Transcendence and Low Interpersonal Communication were fully mediated by Social Support. While both showed significant associations in the initial model (Low Self-Transcendence: *β* = 0.103, *p* < 0.01; Low Interpersonal Communication: *β* = −0.121, *p* < 0.001), their direct effects became non-significant after incorporating Social Support. This indicates their relationship with Social Responsibility operates entirely through the suppression of Social Support: individuals with Low Self-Transcendence struggle to obtain support due to challenge avoidance, while those with Low Interpersonal Communication experience support network deficiencies resulting from social withdrawal. The full mediation pattern for these two dimensions holds particular significance within the Chinese cultural context. In a society that emphasizes “striving” and “relationships,” avoiding challenges and social withdrawal are perceived as more significant deviant behaviors, thus making individuals more reliant on compensation from external support systems.

The total effect of High Dependence was not significant (*β* = −0.009, *p* = 0.779), and its complex mechanism requires further interpretation in conjunction with the “inconsistent mediation” pattern.

#### Group differences: elevated “Buddha-like” risk among undergraduate students

Demographic analysis indicated that undergraduate students demonstrated significantly higher scores than vocational college students in Low Goal Commitment (*t* = 2.83, *p* < 0.01) and Low Interpersonal Communication (*t* = 3.52, *p* < 0.001). This counterintuitive finding may stem from differing educational emphases: undergraduate education, with its theoretical focus, exposes students to dual uncertainties regarding further education and employment, potentially leading to “goal suspension.” In contrast, vocational education emphasizes practical skills, offers relatively clearer career pathways, and incorporates teamwork-based courses that facilitate interpersonal communication. Therefore, “Buddha-like” characteristics are not confined to perceived “disadvantaged groups” but also warrant significant attention among undergraduate students, often considered “elite reserves.”

This phenomenon must also be understood within the context of China’s educational culture. Unlike vocational education systems in countries like Germany that emphasize diversified development, undergraduate education in China carries the heavy burden of familial expectations and its function as a vehicle for social mobility. When reality falls short of these expectations, students are more susceptible to entering a state of “goal suspension.” In comparison, the clear skill orientation and relatively less pressured expectation environment of vocational education provide students with more concrete anchors for achievement.

### Special psychological mechanisms: revelation of suppression effect and inconsistent mediation

Through fine-grained analysis of the various dimensions of “Buddha-like” characteristics, this study identified two important complex statistical phenomena—suppression effects and inconsistent mediation, manifested in the dimensions of Low Self-Transcendence and High Dependence, respectively. Together, they delineate the multiple pathways through which “Buddha-like” characteristics are associated with Social Responsibility.

#### The suppression effect of low self-transcendence: the masked negative nature

This study found that the relationship between Low Self-Transcendence and Social Responsibility exhibited a typical suppression effect. After controlling for other “Buddha-like” dimensions, its regression coefficient changed from negative to positive (*β* = 0.103, *p* < 0.01), suggesting potential confounding by other variables. Introducing Social Support as a mediator fundamentally altered this pathway: the direct association of Low Self-Transcendence with Social Responsibility reversed from positive to negative and became non-significant (*β* = −0.014, *p* = 0.598), consistent with the statistical suppression effect pattern defined by MacKinnon et al. (2000).

These results indicate that Social Support acts as a key suppressor variable, potentially masking the underlying negative association between Low Self-Transcendence and Social Responsibility. The intrinsic mechanism appears to involve a form of “passive support dependence” among individuals with low self-transcendence. While superficially demonstrating positive reliance on social support (Family Support: *β* = 0.149; Friend Support: *β* = 0.238; Other Support: *β* = 0.172), this reliance is characterized by maintaining shallow, low-investment social relationships to avoid challenges and risks, rather than actively constructing high-quality support networks. Consequently, after accounting for the role of Social Support, the negative aspects inherent in Low Self-Transcendence—such as challenge avoidance and lack of initiative—become more apparent. Ultimately, Low Self-Transcendence is linked to lower Social Responsibility indirectly through the fully mediated pathway of Social Support. This finding is also consistent with the social cognitive theory framework concerning how individual traits relate to behaviors through environmental resources.

Therefore, the suppression effect exhibited by the Low Self-Transcendence dimension reveals the deep motivational conflicts potentially hidden beneath its surface attitude of “going with the flow.” Within the context of China’s mainstream culture that champions “striving” and “self-transcendence,” individuals may unconsciously maintain a form of “passive support dependence,” thereby covertly fulfilling their actual motivation to avoid challenges without overtly violating cultural norms. Social Support plays the role of a “suppressor variable” precisely because it is a highly encouraged and commended resource within Chinese culture.

#### The inconsistent mediation of high dependence: a context-dependent dual-pathway

Bootstrap tests indicated that the total effect of High Dependence on Social Responsibility was not significant (effect value = −0.008, *p* = 0.779). However, its indirect effect through Family Support reached statistical significance (effect value = 0.027, 95% CI [0.001, 0.061]), while the direct effect was negative (effect value = −0.042, *p* = 0.07), constituting a typical inconsistent mediation pattern (MacKinnon et al., 2007).

This result reflects a “context-dependent” dual mechanism in how High Dependence relates to Social Responsibility. In family contexts, individuals with high dependence, due to their “compliant and non-refusing” traits, may more readily accept parental responsibility norms and expectations, thereby exhibiting an indirect positive association with Social Responsibility through enhanced Family Support. However, in broader social contexts, the tendencies associated with this trait—such as “lack of assertiveness and weak principles”—may be linked to evading responsibility or blind conformity under group pressure, thus showing a direct negative association with Social Responsibility. The positive indirect path and the negative direct path counterbalance each other, resulting in a non-significant total effect. This finding reveals that High Dependence is not a unidimensional negative trait; its effects are highly dependent on the nature of support and behavioral demands within specific social contexts.

## Limitations and future research directions

This study has several limitations that also delineate clear avenues for future inquiry. While providing valuable empirical evidence for understanding the relationships between college students’ “Buddha-like” characteristics and Social Responsibility, including the mediating role of Social Support, the following constraints should be acknowledged.

Primarily, the most significant limitation stems from the cross-sectional research design. Although the mediation model is grounded in social cognitive theory, the data themselves cannot support causal inference. Potentially complex bidirectional relationships may exist among “Buddha-like” characteristics, Social Support, and Social Responsibility. For example, lower levels of Social Responsibility might be associated with strengthened “Buddha-like” tendencies, while sufficient Social Support could be related to a reduction in the “Buddha-like” mindset. Future studies should employ longitudinal designs with multiple time points to elucidate the dynamic interplay and temporal sequencing among these variables. Constructing competing models to examine reverse pathways, such as “Social Responsibility → “Buddha-like” characteristics,” would also help assess the robustness of the proposed model structure. Furthermore, experimental or intervention research could more directly investigate whether efforts to enhance Social Support are associated with mitigating “Buddha-like” characteristics and fostering Social Responsibility.

Secondly, this study has certain limitations at the measurement and model levels. Although the overall theoretical model demonstrated an acceptable fit to the data (with satisfactory indices such as RMSEA and SRMR), some relative fit indices (e.g., CFI and TLI) were slightly below the excellent standard of 0.90. This might be attributable to the model’s complexity and the large sample size. Future research could attempt to optimize the model specification within theoretical constraints. Simultaneously, the Average Variance Extracted (AVE) for the “High Dependence” dimension was below the recommended threshold of 0.50, indicating room for improvement in the measurement of this construct. Although this dimension was retained due to its theoretical importance, this limitation suggests that future studies may need to revise or develop new items to measure “High Dependence” more precisely.

Third, the generalizability of our findings may be limited by the sampling strategy. Despite the relatively large sample size, the data originated from a convenience sample of Chinese universities. Given that the “Buddha-like” phenomenon is culturally situated and intertwined with China’s specific educational context, the applicability of our conclusions to other populations (e.g., working young adults, secondary school students, or students from other cultural backgrounds) may be constrained. Future research should test the applicability and stability of this model across more diverse populations and cultural settings.

Fourth, despite implementing procedural controls and statistical remedies to address common method bias, the use of self-report data may still introduce subjective biases. This is particularly pertinent for measuring constructs like Social Responsibility, which are susceptible to social desirability effects. Future investigations could benefit from employing multi-method, multi-source data collection strategies. Incorporating peer ratings, teacher observations, behavioral data, or situational experiments would allow for a more comprehensive and objective assessment of “Buddha-like” characteristics and Social Responsibility.

Fifth, this study did not account for potential psychological confounding variables such as general self-efficacy, neuroticism, and social desirability. Although key demographic variables were included in the model and hypotheses were derived from social cognitive theory, these unmeasured traits might correlate with “Buddha-like” characteristics, Social Support, and Social Responsibility, potentially influencing the accuracy of the estimated mediation effects. Future work should systematically incorporate these variables to evaluate the robustness of the Social Support mediation pathway and clarify the unique explanatory power of “Buddha-like” characteristics.

Building upon these limitations and the current findings, future research could explore the following directions:

**Interventions targeting the “Buddha-like” mindset and social support.** Develop and evaluate targeted intervention programs, such as mental health and stress management courses, to help students identify stressors underlying “Buddha-like” characteristics and explore their connections to goal setting and initiative. Enhancing social practice and teamwork within curricula could examine their relationships with social skills and “Buddha-like” traits. Designing personalized development plans might be associated with stimulating intrinsic motivation. Establishing multi-level support systems, including family education guidance and improved teacher-student interactions, could be evaluated for their connections to reducing “Buddha-like” characteristics and promoting Social Responsibility. Future intervention studies can not only test the local effectiveness of these programs but also provide unique cases and solutions from Eastern culture for global practices addressing youth motivation loss and social alienation.

**Deepening the mechanisms linking social support and social responsibility.** Utilize experimental methods to examine whether enhancing Family Support (e.g., through parental emotional responsiveness training) is associated with increased Social Responsibility. Evaluate how peer support programs (e.g., cooperative learning, club activities) relate to interaction quality and Social Responsibility. Investigate the connections between teacher-student mentoring relationships and the development of Social Responsibility both within and beyond the classroom. More importantly, by investigating different types of social support, future research can rigorously test whether the “dominant role of Family Support” mechanism identified in this study holds in individualistic cultures, thereby clarifying the cultural boundary conditions of social support functions and providing cross-cultural evidence for developmental psychology and social cognitive theory.

**Integrating diverse pathways for value guidance.** Explore effective methods for integrating core values such as the spirit of striving and collective consciousness into curriculum systems. Leverage new media platforms to disseminate positive role models and assess their relationships with mitigating the negative influences of “Buddha-like” culture and reshaping value judgments. Through practical approaches like university-industry collaboration and community service, examine how authentic experiential learning relates to the development of Social Responsibility and professional attitudes. This exploration will address a core issue in global youth studies: how to effectively transmit values in different cultural contexts during the post-materialist era to counteract prevalent nihilism and low-desire tendencies.

In conclusion, acknowledging these limitations—pertaining to the cross-sectional design, model fit and measurement, sample representativeness, method biases, and unaccounted confounding variables—is not intended to diminish the value of this study but to foster more rigorous advancement in this field. Systematically addressing these challenges will pave clearer research avenues for a deeper understanding of youth “Buddha-like” characteristics and their mechanisms of social participation, thereby enhancing comprehension of this youth cultural phenomenon and its complex relationship with social engagement.

## Data Availability

The raw data supporting the conclusions of this article will be made available by the authors, without undue reservation.

## Appendix

Appendix A. Dimensional Structure and Sample Items of the Scale of College Students’ “Buddha-like” Characteristics

This study adopted the Scale of College Students’ “Buddha-like” Characteristics developed by Xu and Liu (2022), which consists of 4 dimensions and 20 items. The scale uses a 5-point Likert scoring system (1 = “Completely Inconsistent” to 5 = “Completely Consistent”). The higher the average score of each dimension (calculated by summing and averaging the scores of items under the dimension), the more prominent the “Buddha-like” characteristics of that dimension.

The operational definitions and sample items of each dimension are presented below (2 representative items are listed for each dimension):

A1 Low Goal Commitment

**Definition:** It refers to the state where an individual lacks the behavior of persisting in and pursuing goals, manifested as insufficient motivation, procrastination in handling affairs, vague goals, and avoidance of investment.

Sample Items:

In life and study, I do not have very clear goals and just muddle along.

I mostly hold an indifferent attitude towards doing things.

A2 Low Self-Transcendence

**Definition:** It refers to the state where an individual lacks the willingness to challenge themselves and break through the current situation, manifested as avoidance of competition, being content with stability, low ambition, and a laissez-faire mindset.

Sample Items:

I prefer a comfortable and stable life.

I do not have much ambition and set low requirements for myself.

A3 Low Interpersonal Communication

**Definition:** It refers to the state where an individual shows a withdrawn tendency in social interactions, manifested as poor communication skills, preference for being alone, neglect of expressing themselves, and avoidance of collective activities.

Sample Items:

I do not like interacting with others very much and prefer to be alone.

I rarely participate in group activities on or off campus.

A4 High Dependence

**Definition:** It refers to the state where an individual lacks autonomy and a sense of principles in interpersonal interactions, manifested as difficulty in refusing others, a tendency to conform, vague principles, and avoidance of expressing objections.

Sample Items:

I rarely refuse when others assign tasks to me.

I do not have very firm principles when doing things.
